# Hereditary Vice

**Published:** 1875-03

**Authors:** 


Hereditary Vice.—Some curious statistics regarding inheritance have recently been published by Dr. Harris, of New York. His attention, says the *American Medical Weekly*, was called some time since to a county on the upper Hudson, which showed a remarkable proportion of crime and poverty to the whole population—four hundred and eighty of its forty thousand inhabitants being in the alms-house—and upon looking at the records a little he found certain names continually appearing. Becoming interested in the subject he concluded to search the genealogies of these families, and, after a thorough investigation, he discovered that from a young girl named "Margaret," who was left adrift, nobody remembers how, in a village of the county, seventy years ago, and, in the absence of an alms-house, was left to grow up as best she could have descended two hundred criminals. As an illustration of this remarkable record, in one single generation of her unhappy line there were twenty children; of these, three died in infancy, and seventeen survived to maturity. Of the seventeen, nine served in the State prisons for high crimes an aggregate term of fifty years, while the others were frequent inmates of jails and penitentiaries and alms-houses! The whole number of this girl's descendants, through six generations, is nine hundred; and besides the two hundred who are on record as criminals, a large number have been idiots, imbeciles, drunkards, prostitutes and paupers. A stronger argument in favor of the inheritance of vice, and for careful treatment of pauper children, could hardly be found.—*Boston Med. and Surg. Jour.*, Jan. 28, 1875, p. 112.

